# Long Noncoding RNAs in Yeast Cells and Differentiated Subpopulations of Yeast Colonies and Biofilms

**DOI:** 10.1155/2018/4950591

**Published:** 2018-03-25

**Authors:** Derek Wilkinson, Libuše Váchová, Otakar Hlaváček, Jana Maršíková, Gregor D. Gilfillan, Zdena Palková

**Affiliations:** ^1^Faculty of Science, Charles University, BIOCEV, 252 50 Vestec, Czech Republic; ^2^Institute of Microbiology of the Czech Academy of Sciences, BIOCEV, 252 50 Vestec, Czech Republic; ^3^Department Medical Genetics, Oslo University Hospital and University of Oslo, 0450 Oslo, Norway

## Abstract

We summarize current knowledge regarding regulatory functions of long noncoding RNAs (lncRNAs) in yeast, with emphasis on lncRNAs identified recently in yeast colonies and biofilms. Potential regulatory functions of these lncRNAs in differentiated cells of domesticated colonies adapted to plentiful conditions versus yeast colony biofilms are discussed. We show that specific cell types differ in their complements of lncRNA, that this complement changes over time in differentiating upper cells, and that these lncRNAs target diverse functional categories of genes in different cell subpopulations and specific colony types.

## 1. Introduction

*Saccharomyces cerevisiae* strains used in the brewing industry and in microbiology and genetics laboratories are often grown as planktonic cells in liquid culture, but yeasts also form multicellular communities such as colonies and biofilms, which reflect a more natural lifestyle and are able to cope with different intrinsic and extrinsic stresses [1]. There is growing evidence of cell differentiation, metabolic reprogramming, activation of various stress-defence mechanisms, and other aspects of primitive multicellularity, not only in the complex colony biofilms of nutritionally challenged wild yeast but also in the less structured, smooth colonies of pampered laboratory strains [2–5]. Ammonia signalling, metabolic reprogramming, mitochondrial retrograde signalling, the presence of extracellular matrix, chromosome rearrangement, and many other processes have been described that contribute towards the colony lifestyle, differentiation processes, stress resistance, adaptation, and longevity of multicellular populations [1, 3–7]. However, lncRNA has, until recently, been overlooked as a potential regulator of processes involved in long-term colony development and differentiation, despite the key roles of regulatory ncRNAs in mammalian cell differentiation [8]. The RNAi machinery, which contributes to the production of regulatory ncRNA in many organisms, has been lost in *S*. *cerevisiae* [9]. Studies in yeast [10–12] identified large numbers of “cryptic transcripts,” “nonannotated transcripts,” and “heterogenous unstable RNAs,” respectively. These studies established the use of tiling arrays for the identification of yeast long noncoding RNA (lncRNA) and deletion of genes encoding exonucleases, such as *RRP6*, to stabilise unstable transcripts. Loss of RNAi machinery may have triggered the evolution of a large complement of highly expressed lncRNA in yeast [13]. The detection of several thousand lncRNAs in two studies [14, 15] led to an explosion of interest in these poorly understood transcripts.

**Figure 1:**
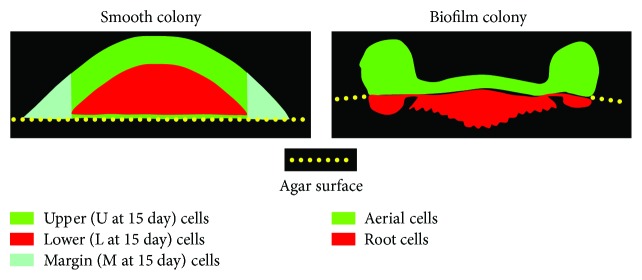
Diagram of cell subpopulations isolated from smooth colonies of BY4742 strain (a) and biofilm colonies of BR-F strain (b).

Here, we present a mini review of yeast lncRNAs and their previously described roles in regulating gene expression under various circumstances. In the second part of the review, we focus in more detail on lncRNAs that we have recently identified in differentiated subpopulations of cells from two distinct types of yeast populations [16, 17], each of which uses unique strategies to cope with stress and ensure longevity of the population as a whole. These are complex colony biofilms formed by wild strains of *Saccharomyces cerevisiae* [18] and smooth colonies of *S. cerevisiae* laboratory strains [19] (Figure 1). We present further analyses of these lncRNAs, particularly in relation to their different types and positions in relation to neighbouring genes. We also discuss potential regulatory activities of lncRNAs in ageing smooth colonies and colony biofilms in light of current knowledge of regulatory functions of lncRNAs in yeast cells.

## 2. Important Messages or Random SPAM?

Transcription of yeast lncRNA occurs largely from bidirectional promoters shared with other loci [10, 14, 20–23]. However, lncRNA accumulation is countered by early termination of unstable antisense transcription, modulation of strand expression via chromatin remodelling, and degradation of lncRNAs [14, 15, 22, 24]. lncRNA/gene expression correlation [25, 26] suggests that some lncRNAs are true cellular regulators. Furthermore, there are numerous examples of the stabilisation (or destabilisation) of lncRNA transcript classes under specific conditions, such as meiosis, respiration or sporulation [26–29], carbon source [14, 30], metal abundance [31], and osmotic stress [32]. It was recently shown that the 5′–3′ exonuclease Xrn1p is localised to eisosomes when glucose is scarce but relocalises to the cytoplasm when glucose is present, where it degrades lncRNAs called XUTs and modulates lncRNA regulation of gene expression [33]. Whether this phenomenon constitutes primary regulation or merely “fine-tuning” of gene expression remains to be determined. Nonetheless, it is clear that the study of lncRNA in yeast may uncover important regulatory mechanisms. On the other hand, some lncRNA transcription may simply be a by-product of bidirectional transcription [15].

## 3. Classes of lncRNA: Stability and Detection

The identification of different classes of lncRNAs in yeast has been largely determined by the techniques used in their detection. Microarrays, 3′-long serial analysis of gene expression (SAGE), and *RRP6* deletion were used to identify stable unannotated transcripts (SUTs), lncRNAs that are processed in the cytosol similarly to mRNAs, and cryptic unstable transcripts (CUTs) that are sensitive to the RNA decay machinery and degraded by the nuclear exosome and/or the cytoplasmic 5′–3′ exonuclease Xrn1p [14, 15, 34]. Other lncRNAs follow this stability-based nomenclature (Table 1), which will also be used in this text. MUTs (meiotic unstable transcripts) are a subset of CUTs that are degraded by Rrp6p RNase (a component of the nuclear exosome complex (NEC)) which accumulate predominantly during meiotic development due to decreased levels of Rrp6p [28]. rsCUTs are expressed during respiration and/or sporulation [28]. XUTs (Xrn1p-sensitive unstable transcripts) are another class of CUTs that are degraded in the cytoplasm. Deletion of *XRN1*, which encodes a 5′–3′ exonuclease, stabilises XUTs [34]. Reducing Nrd1p levels in the nucleus inhibits Nrd1p-dependent lncRNA transcription termination [22], allowing identification of Nrd1-unterminated transcripts (NUTs). Telomeric repeat-containing RNA (TERRA), regulating telomere function, is stabilised in *rat1-1* mutants which are defective in 5′–3′ nuclear exonuclease activity [35]. *NAM7* (*UPF1*) encodes an RNA helicase involved in the nonsense-mediated decay (NMD) pathway, and deletion of *NAM7* facilitates accumulation of 5′-extended transcripts which, because their accumulation depends on inactivation of cytoplasmic degradation, were termed “cytoplasmically degraded CUTs” (CD-CUTs) [31].

Termination of CUT transcription occurs via a different mechanism from that of mRNAs [36]. The Nrd1p-Nab3p complex, which includes helicase and cap-binding proteins, binds specific motifs that are enriched in CUTs and targets nascent lncRNAs for adenylation and degradation via interactions with the TRAMP complex and nuclear exosome [36–39]. CUTs tend to be degraded in the nucleus and are largely sequestered from the cytoplasm under natural conditions, but other classes of lncRNA are exported from the nucleus and degraded in the cytoplasm [39]. Some lncRNA (e.g., CD-CUTs) may even be imported back into the nucleus, following NMD, where they repress gene expression in trans [31, 39]. Once lncRNAs are exported from the nucleus, they may be targeted by several decay pathways [40], including (i) deadenylation, decapping, and degradation by the 5′–3′ exonuclease Xrn1p; (ii) deadenylation, followed by 3′–5′ degradation by the cytoplasmic exosome or via one of the translation-associated pathways; (iii) the nonsense-mediated decay pathway (degrading mRNA containing, e.g., spurious stop codons); and (iv) no-go decay (NGD: degrading mRNA with stalled elongation). Wery et al. [40] review the high degree of overlap between lncRNA classes (e.g., between XUTs and both SUTs and CUTs) and suggest that many XUTs are merely SUTs that have been extended at the 3′ end and that these extensions target XUTs for NMD.

## 4. Regulation of Gene Expression by Annotated lncRNAs

**Figure 2:**
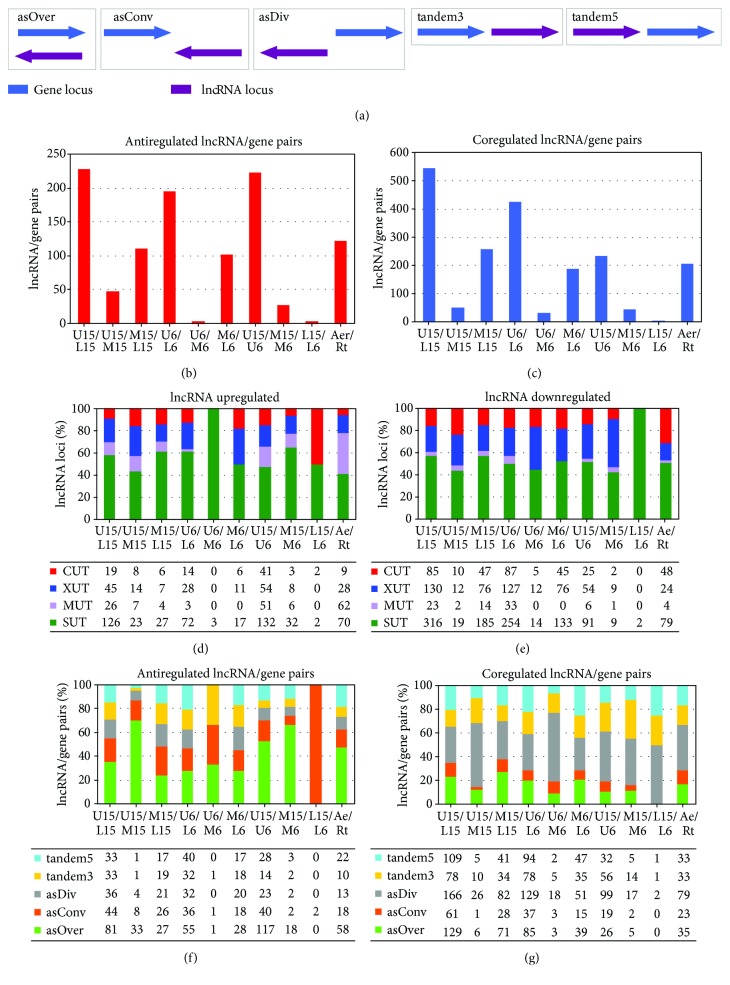
lncRNAs detected in subpopulations of aged yeast colony cells. DE gene and DE lncRNA loci were identified that were located within 1.5 kB of one another in 5 different orientations (a). The numbers of antiregulated (b) and coregulated (c) lncRNA/gene pairs were compared for the different expression comparisons detailed at the bottom of the figure. Percentages (bars) and numbers (boxes underneath) of different classes of lncRNA upregulated (d) or downregulated (e) in each sample as well as the percentages (bars) and numbers (boxes) of antiregulated (f) and coregulated (g) lncRNA/gene pairs are given for each comparison. Abbreviations: asOver: antisense-overlapping; asConv: antisense-convergent; asDiv: antisense-divergent; tandem3: tandem to (sense strand) and downstream of gene; tandem5: tandem to (sense strand) and upstream of gene; SUT: stable unannotated transcript; MUT: meiotic unstable transcript; XUT: Xrn1-dependent unstable transcript; CUT: cryptic unannotated transcript.

lncRNAs differ in terms of distance from coding regions and orientation with regard to coding genes (Figure 2(a)). Antisense transcripts are transcribed from overlapping loci, located on the opposite strand to sense loci [26] (antisense-overlapping). lncRNA/gene pairs on opposite strands may be transcribed from nearby start points but in opposite directions (antisense-divergent orientation), or their transcription may converge on a common end point (antisense-convergent orientation). Alternatively, the lncRNA locus may occur on the same strand as an upstream or downstream locus (tandem sense orientation) [14].

The functions of most yeast lncRNAs identified so far are unknown, but there are some exceptions, including regulation of *GAL1*, *PHO5*, and *PHO84* expression [23], in which the lncRNA has been assigned a role in regulating the expression of a related gene. In some of these examples, the regulatory function is attributed to the transcription process itself and not to the presence of the lncRNA transcript [39]. For example, lncRNA transcription could be involved in changes in chromatin structure that subsequently influence the binding of transcription factors to promoter regions of the related gene, as was shown for the negative regulation of *IME1* [29] and modulation of expression of *FLO11* [41]. Other known mechanisms of lncRNA function include transcriptional interference in which antisense lncRNA can block/decrease transcription of sense-strand mRNA, for example, in the case of *IME4* mRNA transcription [29]. Many lncRNAs are regulated by the same promoter as a divergent gene on the opposite strand [42], and the lncRNA negatively regulates the expression of an antisense-overlapping gene that lies upstream of the antisense-divergent gene.

Expression of the *FLO11* gene, involved in many processes including colony biofilm formation, is modulated by the expression of a tandem upstream sense locus, *ICR1*. *ICR1* expression is in turn regulated by an antisense-overlapping locus, *PWR1* [41] in a “toggle”-like manner, dependent on two transcription factors: Sfl1p activates *ICR1* expression, inhibiting *FLO11* expression, while Flo8p upregulates *PWR1* expression, inhibiting *ICR1* expression and promoting *FLO11* expression [41]. Rpd3p-mediated chromatin modification may block Sfl1p binding, countering *ICR1*-mediated inhibition of *FLO11* expression. Bumgarner et al. [43] revealed the existence of three expression states within a clonal cell population in which the *FLO11* promoter is (i) silenced, (ii) nonsilenced but lacking transcription factors, and (iii) nonsilenced and bound by transcription factors leading to *FLO11* expression that is absent, low, and high, respectively. *FLO11* expression correlated with *PWR1* expression and anticorrelated with *ICR1* expression and the physical act of *ICR1* transcription displaced transcription factors from the *FLO11* promoter, effectively “resetting” the promoter. Cells with fewer than 5 fluorescent Flo11p dots lacked structured colony morphology, diploid pseudohyphae formation, and haploid adhesion. Since Flo11p drives biofilm formation and biofilm development is a key fungal virulence factor in pathogenic fungi such as *Candida albicans* [44–46], identifying regulators of *FLO11* expression could help to counter the high drug resistance of fungal biofilms or uncover drug targets for treatment of potentially lethal fungemias.

In haploid cells, Rme1p binds upstream of the key meiotic regulator *IME1*, causing the tandem sense locus *IRT1* to express a transcript that recruits histone remodellers that produce a repressive chromatin structure over the *IME1* promoter [29]. In MATa/*α* diploids, the a1-*α*2 repression complex blocks *RME1* expression, relieving inhibition of meiosis. Another major regulator of meiosis is *IME4*, encoding a protein that methylates many key sporulation mRNAs [27, 29]. *RME2*, an antisense lncRNA locus overlapping the whole *IME4* ORF, inhibits *IME4* expression in haploid cells. However in diploid cells, the a1-*α*2 complex binds to the *RME2* promoter, inhibiting lncRNA expression and relieving the block on *IME4* expression [29]. In another example of gene expression regulation via chromatin remodelling, the osmotic stress-induced MAPK, Hog1p binds the 3′ end of *CDC28* (a master regulator of mitosis and meiosis), promoting antisense lncRNA expression that triggers looping of the *CDC28* gene. This looping allows Hog1p to “jump” across the narrow neck of the loop from the 3′ to the 5′ end, where it induces RSC-dependent chromatin remodelling, leading to *CDC28* gene expression [32]. These examples demonstrate how lncRNA regulatory mechanisms can be switched on or off in specific cell subpopulations or in the presence of stress [27, 32].

In some cases, transcription of a lncRNA directly hinders gene expression from a neighbouring target gene [39]. When serine is plentiful, expression from a 5′ tandem sense lncRNA locus, *SRG1*, inhibits expression of the serine biosynthesis gene *SER3* by increasing promoter nucleosome density, which prevents transcription factors from accessing the promoter [47]. Martens et al. [48] showed that serine availability activates binding of the transcription factor Cha4p to the *SRG1* promoter and recruitment the Swi/Snf and SAGA complexes, leading to activation of *SRG1* and thus repression of *SER3*. Such “transcriptional interference” is a common mechanism by which the expression of one locus is attenuated by that of a second, converging locus [49], not only between coding and noncoding loci but also between converging coding loci.

A *GAL10* antisense-overlapping lncRNA (which also sense-overlaps *GAL1*) has been shown to recruit methyltransferase and deacetylase complexes which silence both *GAL10* and *GAL1* [39]. Pinskaya et al. [50] showed that glucose/Reb1-dependent transcription of the antisense transcript GAL1_uncut_ promotes Set1-dependent H3K4 di/trimethylation and facilitates recruitment of the RPD3S histone deacetylase, repressing *GAL1*. A similar mechanism blocks transcription from a “hidden” promoter within *SUC2*, suggesting that H3K4 di/trimethylation might represent a widespread mechanism for maintaining promoter fidelity. lncRNAs are decapped and degraded in a *DCP1-*, *DCP2-*, *XRN1-*, and *RAT1*-dependent manner [51] to facilitate galactose-induced GAL expression. Lenstra et al. [52] showed that lncRNA expression has two modes: spurious and functional. When GAL expression is induced, GAL10 expression is independent of spurious lncRNA expression, but when not induced, tight repression of GAL10 is dependent upon functional lncRNA expression. Cloutier et al. and Beck et al. [53–56] demonstrated that the glucose-dependent DEAD box RNA helicase negatively regulates formation of DNA/RNA hybrid R-loops and that a change of carbon source from glucose to galactose promotes export of Dbp2p to the cytoplasm and lncRNA-dependent R-loop formation and displacement of the Cyc8-Tup1p corepressor from the promoter and derepression of GAL genes. Thus, relieving the block on R-loop formation may be a general mechanism for rapidly derepressing key genes and adapting to changing environmental conditions [55]. Zacharioudakis and Tzamarias [57] further showed that when galactose concentration is high, Gal1p enters a positive feedback loop with Gal4p and the GAL genes are turned on, independently of lncRNA. However, when galactose levels are low, the lncRNA is able to randomly block transition to the on state, delaying the switch to alternative carbon source in a percentage of cells and facilitating metabolic flexibility.

Although most cases of lncRNA regulation identified in budding yeast appear to be *cis*-acting, some examples of *trans*-acting regulators have been described [34, 58, 59]. Reference [34] identifies Xrn1p-sensitive antisense transcripts, some with apparent regulatory roles, and suggests a mechanism whereby XUTs interact with a protein complex to silence target genes and that this activity is promoted by histone H3K4 mono/di-methylation but opposed by histone H3K4 trimethylation. Large numbers of different XUTs are exported from the nucleus and may be degraded by translation-coupled NMD [40], so they may also have posttranscriptional regulatory roles [34]. Ty1 retrotransposon expression is regulated in trans by an antisense CUT [58] which interacts with Ty1, promotes histone deacetylation and Set1-dependent methylation, and effects chromatin silencing. It has been shown [59] that *PHO84* antisense lncRNA is able to act in trans to silence transcription of a second copy of *PHO84* elsewhere in the genome. Silencing of the second *PHO84* copy depends upon a region of homology with the upstream activating sequence and possibly also recruits a silencing complex to the promoter.

A lncRNA overlaps the *PHO5* gene on the antisense strand, and transcription of the lncRNA across the gene promoter plays a role in the activation (not repression) of *PHO5* [60] by increasing the efficiency of histone removal, facilitating access of the polymerase to the TATA box. Bunina et al. [61] showed that starvation-/sporulation-induced expression of the antisense transcript is dependent upon repetitive regions in the 3′UTR and has no effect upon promoter activity but does promote the expression of a long mRNA isoform with enhanced stability.

Chia et al. [62] showed that transcription of an upstream lncRNA, *NDC80*_luti_, represses *NDC80* expression during meiotic prophase by driving Set1-dependent H3K4Me2 and Set2-dependent H3K36Me3 at the *NDC80* promoter, leading to recruitment of the Set3C and Rpd3S histone deacetylases. The pervasiveness of lncRNA regulation of gene expression in different cell types and under differing conditions has been demonstrated in a number of studies. Kim et al. [63] showed that the promoters of a high percentage of Set2-repressed genes are overlapped by antisense or upstream tandem lncRNA that promote H3K36Me3 and Rpd3S-dependent deacetylation within the promoter region and that many of these genes are regulated by carbon source. A similar mechanism involving Set3C-dependent H3K4me2 was identified previously [30]. McDaniel et al. [64] showed that deleting *SET2* affects the expression of genes involved in stress responses because lack of H3K36Me permits the inappropriate transcription of antisense lncRNA that interferes with gene transcription.

Kwapisz et al. [65] identified CUTs and XUTs, generated from subtelomeric regions (subTERRA) with roles in telomeric silencing and prevention of clustering, respectively, via the formation of DNA-RNA hybrids and by protein scaffolding.

## 5. Long ncRNAs May Contribute to Gene Regulation within Differentiated Cell Subpopulations of Colonies and Colony Biofilms

Traven et al. [66] provided a first glimpse into the presence of, and potential regulation of genes by, lncRNAs within yeast colonies of laboratory strain BY4741, grown on complete glucose medium and differentiated into two subpopulations of cells on the “outside” and “inside” of the colonies. In this study, transcriptomic differences were identified by microarrays and included 12 SUTs and CUTs on the outside and 53 on the inside of the colonies. In addition, several lncRNA/gene pairs with positively correlating expression were identified that represent possible examples of gene regulation by lncRNA, including the ammonium permease gene *MEP2*.

We performed further studies using the more sensitive RNA sequencing (RNA-seq) technique. RNA-seq provided a detailed transcriptomic view of six cell subpopulations present in smooth BY4742 colonies grown on complete respiratory medium [17]: cells from upper, margin, and lower parts of colonies in two developmental phases (late acidic 6-day-old and alkali-phase 15-day-old). In parallel experiments, two subpopulations (a surface “aerial” cell subpopulation and a subpopulation of invasive “root” cells growing within the agar) from structured colony biofilms grown on the same medium were also studied [16] (Figure 1). Every gene located within 1.5 kB of (or antisense-overlapping) each lncRNA was identified to produce a list of lncRNA/gene pairs in any of the 5 different orientations (Figure 2(a)) considered. Previous expression profiling of subpopulations of smooth colonies and colony biofilms identified some metabolic similarities but even more differences [16, 17]. Whereas some of the expression differences in individual genes may be caused by the fact that laboratory and wild strains forming smooth colonies and colony biofilms, respectively, are not isogenic, most of the differences are in agreement with the different lifestyles of yeast populations in smooth colonies (formed by either laboratory strains or domesticated wild strains) versus colony biofilms [1, 5]. Here, we therefore also compared types of identified lncRNAs as well as lncRNA/gene pairs in smooth colonies and colony biofilms, to see whether any potential lncRNA-related similarities exist among subpopulations of these structures.

### 5.1. Antiregulation of lncRNA/Gene Pairs Is Highest when Comparing Dissimilar Cell Types

Cells localised to the upper and marginal regions of smooth colonies (Figure 1) are somewhat similar in gene expression, protein production, and so on and are more different from cells localised to the lower regions of central colony areas [17]. lncRNA/gene antiregulation (where gene and lncRNA are antagonistically differentially expressed in the two subpopulations) and coregulation (agonistically differentially expressed) were highest when upper (U) and lower (L) cells were compared and lowest when upper and margin (M) cells were compared (Figures 2(b) and 2(c)). Comparison of aerial and root cells of colony biofilms revealed numbers of antiregulated and coregulated pairs, closest to the numbers identified in marginal/lower cell comparisons in smooth colonies (Figures 2(b) and 2(c)). The numbers of mapped reads were similar for biofilm and smooth colonies (average of 17.1 and 15.4 million reads, resp.); the percentages of mapped reads mapping to lncRNA were 23 and 25%, respectively; and the same read counting and differential expression analysis packages were used in both analyses.

Increased antiregulation and coregulation in the U/L and M/L comparisons, compared with U/M (in both 6- and 15-day-old colonies), is consistent with observed U/M cell similarities (metabolic, gene expression, and other) and differences of both from L cells [17]. High U6/U15 anti- and coregulation agrees with the finding that temporal gene expression changes are most prominent in upper cells of developing colonies [17]. Surprisingly, approximately twice as many antiregulated/coregulated pairs were observed when comparing upper versus lower cells (in smooth colonies) than in root versus aerial parts of biofilm colonies. However, aerial and root parts of colony biofilms are not homogenous and contain small subpopulations of cells with features typical of their counterparts [16]. This fact may dilute the observed aerial-root cell differences. Furthermore, aerial-root cells were separated from younger colony biofilms (3-day-old) than the cells of smooth colonies (6- and 15-day-old), in which upper cells gradually acquire unique metabolic features and gain specific physiology important for longevity [19, 67]. In contrast, only moderate expression changes occur during this time period in slowly growing marginal and lower cells [17]. Accordingly, expression differences between margin and lower cells are more comparable at different developmental time points. However, the aerial versus root and margin versus lower cell comparisons are similar only in terms of the numbers of co- and antiregulated lncRNA/gene pairs, which may reflect merely the level of similarity/dissimilarity between the respective cell types. As shown below, different lncRNA/gene pairs were identified in smooth colonies and colony biofilms.

### 5.2. Cell-Type-Specific Expression of lncRNA Classes

Numbers of differentially expressed lncRNAs differ significantly when comparing the various cell subpopulations (Figures 2(d) and 2(e)). The terms upregulation/upregulate (or downregulation/downregulate) are relative, so the observations could be caused by activation (or repression) of transcription in the first subpopulation or by repression (or activation) in the second. Lower cells were found to upregulate the highest number of lncRNAs of all monitored cell types, as shown in Figures 2(d) and 2(e). Margin cells upregulate 10 times as many lncRNA loci as upper cells at 6 days, whereas no difference was observed at 15 days, which is consistent with the finding that the number of upregulated lncRNAs in upper cells increases over time. No differences in the total number of up-/downregulated lncRNAs were observed between aerial and root cells of colony biofilms.

SUTs were the most common lncRNAs in smooth colonies (>50% of both upregulated and downregulated lncRNAs) as well as in colony biofilms (40% of upregulated and >50% of downregulated lncRNAs). Differentially expressed SUTs were similarly distributed between upregulated and downregulated lncRNA categories across most comparisons (Figures 2(d) and 2(e)). CUTs form ~15% of up-/downregulated genes in most comparisons with no significant difference in distribution in smooth colonies. However, significant differences were detected in colony biofilms, where 5.3x as many CUTs were upregulated in roots (forming 30% of all upregulated lncRNAs in roots) as in aerial cells.

The clearest difference in up- and downregulated unstable lncRNAs between smooth colonies and colony biofilms was based on MUTs, forming ~6.5% and ~20% of up-/down regulated lncRNAs, respectively. More MUTs are up- or downregulated in 15-day-old smooth colonies than in 6-day-old colonies, in which MUTs were only identified when comparing L and U cells (10x as many MUTs were upregulated in L cells), indicating increased MUT expression during smooth colony ageing. The largest group of upregulated MUTs occurred in aerial cells of colony biofilms, >14x more than the number of upregulated MUTs in roots. In summary, expression of MUTs is increased in upper (and partially in margin) parts of smooth colonies during ageing, whereas MUTs are already highly expressed in aerial cells of much younger colony biofilms.

Increased MUT expression in aerial cells of biofilm colonies is consistent with the upregulation of a large group of meiotic genes in aerial cells [16]. MUTs accumulate predominantly during meiotic development, possibly due to decreased levels of Rrp6p RNase (a component of NEC), which can degrade MUTs [28] and CUTs [14, 15]. Accordingly, *RRP6* expression is >2-fold upregulated in root cells than in aerial cells. In smooth colonies, *RRP6* expression (which can affect CUT stability) is only moderately upregulated (1.3- to 1.4-fold) in L relative to U or M cells. However, CUT degradation is also dependent upon Nab3p, Nrd1p, and the TRAMP complex (mainly the TRAMP4 complex, which includes Pap2p (Trf4p) [68]). The apparent upregulation of CUT expression in lower cells and MUT expression in upper cells may be due to differential expression of the TRAMP4 and TRAMP5 complexes (which target both shared and unshared transcripts [69]) and of the 3′–5′ exosome components Rrp6p and Dis3p.

### 5.3. Orientation of lncRNA/Gene Pairs Differs in Antiregulated and Coregulated Pairs

lncRNA/gene pairs were classified according to their mutual position (Figure 2(a)) and expressional relationship (antiregulated, Figure 2(f), and coregulated, Figure 2(g)). The total number of coregulated versus antiregulated lncRNA/gene pairs was slightly higher in both smooth colonies (>1.8x) and colony biofilms (>1.7x), but more prominent differences were observed between specific cell types and among different lncRNA/gene position categories. Coregulated lncRNA/gene pairs were overrepresented as compared with antiregulated pairs in U15/L15 (>2.3x), M15/L15 (>2.3x), U6/L6 (>2.1x), and U6/M6 (>10x) cell comparisons.

Antisense-overlapping (asOver) lncRNAs were the most common category of antiregulated lncRNA/gene pairs both in smooth colonies (>38%) and in colony biofilms (>47%), whereas antisense-divergent (asDiv) lncRNAs were the most prominent category in coregulated lncRNA/gene pairs (>33% in smooth colonies and >38% in colony biofilms). Enrichment of antisense-divergent loci among coregulated and antisense-overlapping loci among antiregulated lncRNA/gene pairs is consistent with previous reports of a positive correlation between the expression of antisense-divergent loci (gene and lncRNA), possibly because of increased bidirectional transcription from a common nucleosome-depleted region [14] and of interference by antisense-overlapping lncRNA in gene expression and thus negative regulation [26]. The distribution of asOver and asDiv lncRNA/gene pairs among different cell comparisons was relatively equal, with the exception of U15/M15 (1.82x more asOver antiregulated pairs than average and 1.65x more asDiv coregulated pairs than average), U6/M6 (1.74x more asDiv coregulated pairs than average), and M15/M6 (1.73x more asDiv coregulated pairs than average) comparisons.

### 5.4. Different Functional Groups of Genes May Be Negatively Regulated by lncRNA in Smooth and Biofilm Colonies

**Figure 3:**
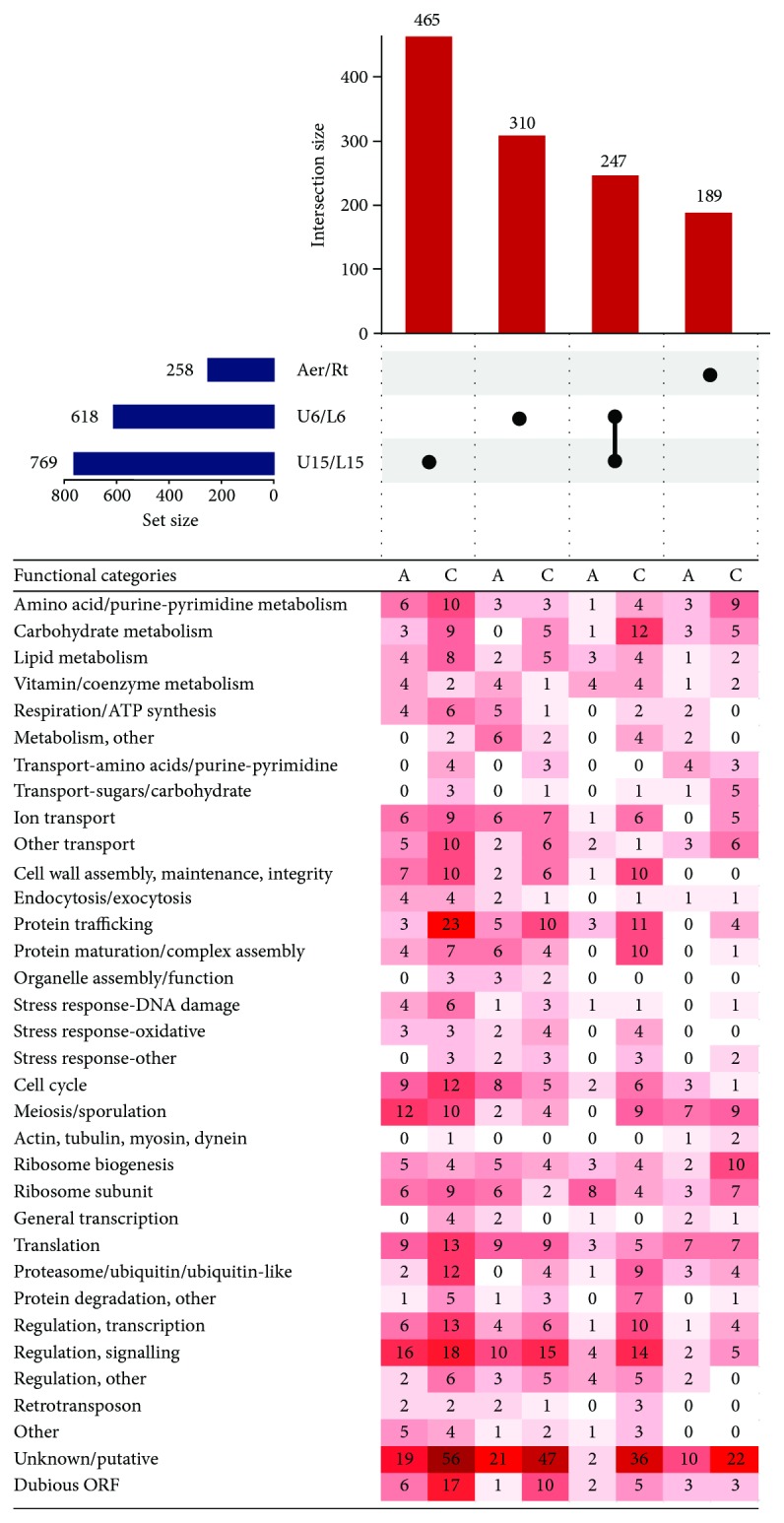
UpSet plot of datasets of coregulated and antiregulated genes in the upper and lower parts of biofilm colonies (Aer/Rt) and of 6-day-old (U6/L6) and 15-day-old (U15/L15) smooth colonies. Coregulated and antiregulated gene/lncRNA pairs in three cell comparisons: aerial versus roots of biofilm colonies, upper cells versus lower cells of 6-day-old smooth colonies, and upper cells versus lower cells of 15-day-old smooth colonies [16, 17]. Horizontal blue bar chart indicates numbers of genes, co-/antiregulated (with lncRNA) in each individual comparison. The black dots and lines (intersect “connectors”) above the heat map indicate comparisons in which the given number of genes (vertical red bar chart) were co-/antiregulated. Only major intersections are shown. Heat map of genes assigned to functional categories and clustered according to functional category (FC) and co-/antiregulated in different sample comparisons (lower part). Number in heat map cell = number of genes from FC, coregulated or antiregulated with lncRNA in a sample comparison. The higher the number of co-/antiregulated genes, the more intense the colour. A: antiregulated; C: coregulated.

The numbers of potentially negatively (in antiregulated lncRNA/gene pairs) and positively (in coregulated lncRNA/gene pairs) regulated genes in different functional annotation groups were considered. Antiregulated/coregulated lncRNA/gene pairs were annotated with functional categories using information in SGD (http://www.yeastgenome.org/, [70]) and the literature. Datasets of differentially expressed (DE) genes were then compared using Intervene's UpSet module [71], which visualizes the intersection of multiple data sets in UpSet plots (Figure 3). No common antiregulated and only 6 common coregulated lncRNA/gene pairs were identified in U15 versus L15, U6 versus L6, and aerial versus root cell comparisons, and these include the genes *KSP1*, *PRC1*, *YNL200C*, *LDS2*, *RRT8*, and *NCR1*, encoding a serine/threonine phosphatase with a putative role in TOR signalling, a vacuolar carboxypeptidase Y, a NADHX epimerase, 2 paralogous spore wall assembly proteins, and a vacuolar membrane protein involved in sphingolipid metabolism, respectively. Over 14% of antiregulated lncRNA/gene pairs in upper versus lower cells are shared between 6-day- and 15-day-old smooth colonies, but only 1% is shared by smooth colonies and colony biofilms (6- or 15-day-old). There are major morphological, expression, and metabolic differences between the two colony types [5, 18, 19], the biofilm colony strain BR-F is diploid while the smooth strain BY4742 is haploid, and signalling and coding RNA expression differences between aerial and root cells may outweigh differences in gene expression regulation by lncRNA.

465 lncRNA/gene pairs were coregulated (308) or antiregulated (157) exclusively in U15 versus L15 cells (Figure 3), including antiregulated genes with roles in regulation/signalling, meiosis/sporulation, cell cycle, translation, and cell wall assembly/maintenance/integrity (Figure 3). 310 pairs were coregulated (184) or antiregulated (126) exclusively in U6 versus L6 cells, including antiregulated genes with roles in regulation/signalling, translation, cell cycle, and ribosomal biogenesis as well as those encoding ribosome subunits (Figure 3). These data suggest that development of upper cells may be partially dependent upon the negative expression regulation (by antiregulated lncRNA) of genes with roles in processes such as the mitotic exit network (*AMN1*, *DBF2*, and *NUD1*), bud site selection (*GIC1*, *RSR1*, and *RAX1*), cytokinesis (*AIM44*), mitotic transitions and checkpoints (*SWE1*, *SPC25*, and *HSL1*), Ras signalling (*IRA1* and *BMH2*), glucose signalling (*YCK1* and *YAK1*), and mating signalling (*DIG2*, *MF(ALPHA)2*, and *PPQ1*). 247 pairs were coregulated (198) or antiregulated (49) in both U15/L15 and U6/L6 cell comparisons but not in the aerial-root cell comparison (8 of the antiregulated genes in this group encode ribosome subunits; the others are dispersed among many functional categories). 189 pairs were coregulated (122) or antiregulated (67) exclusively in the colony biofilm cell comparison, including antiregulated genes with roles in meiosis/sporulation and translation (7 each).

In the time point comparisons, 222, 27, and 2 lncRNA/gene pairs, respectively, were antiregulated in the U15/U6, M15/M6, and L15/L6 comparisons. The fact that 89% of these pairs (222 of 251) were antiregulated in upper cells (U15/U6 comparison) suggests that lncRNA regulation of gene expression changes most during development of U cells. This is consistent with the finding that most of the temporal gene-expression changes occur in upper cells, whereas temporal changes in lower cells and, in particular, the margin cell are moderate [17]. Genes encoding ribosome subunits, or involved in ribosome biogenesis or translation, typically appear DE together with neighbouring lncRNA, and while there is some degree of lncRNA/gene pair overlap between 6-day- and 15-day-old colonies, many genes seem to be selectively regulated in 6-day- or 15-day-old colonies. The translation initiation factor gene *TIF1* is upregulated, while its lncRNA is downregulated in U and M relative to L cells in 15-day-old colonies only, suggesting that differentiation may require divergence in fine-tuning of translation rates as colonies age. While *RPL36B* is upregulated (and its lncRNA downregulated) in U and M cells, relative to L cells, of both 6-day- and 15-day-old colonies, repression of its paralog *RPL36A* is relieved (potentially by lncRNA downregulation) only in 15-day-old colonies. Since deletion of the latter decreases fermentative growth but increases respiratory growth, its increased expression as the colony ages is consistent with the utilization by U cells of L cell-derived hexoses in differentiated colonies [72]. Stoichiometric changes in the ribosome subunit make-up may thus represent one aspect of the metabolic remodelling program as cells in ageing colonies differentiate.

Cell cycle progression is regulated under stress conditions by antiregulated noncoding RNAs [73] for genes such as *FAR1* (encoding an inhibitor of cyclin-dependent kinase (CDK) Cdc28p involved in cell cycle phase transitions). In addition, Lardenois et al. [28] suggested that expression of the *CLN2* gene that encodes cyclin G1 may be negatively regulated by promoter-overlapping MUT1465 expression, relieving *CLN2*-dependent repression of *IME1* and allowing meiosis to proceed. In colony biofilms, we observed *FAR1* expression to be upregulated 2.8-fold in aerial cells, and its antisense SUT locus SUT204 was upregulated 1.7-fold in roots. Thus, the Far1p CDK inhibitor may elicit mitotic cell cycle arrest in aerial cells, whereas its expression is repressed in roots to permit cell cycle progression. Furthermore, the cyclin *CLN3* is upregulated 1.7-fold in roots and its antisense-overlapping partner MUT30.1 is upregulated 2.1-fold in aerial cells. Similarly, *CLN2* is upregulated 1.7-fold in roots, and the expression of MUT1465.2, which is located upstream (i.e., over the presumed promoter region) of *CLN2*, is upregulated 3.3-fold in aerial cells. These findings are consistent with previous findings that aerial cells have entered the stationary phase in 40-hour-old colonies, whereas root cells continue to divide [18], indicating that lncRNAs may participate in the regulation of cell division in biofilm colony cell subpopulations. The situation in smooth colonies is less clear because MUT30.1 and MUT1465.2 were not detected in smooth colonies and SUT204 is upregulated 3-fold in L relative to U cells and *FAR1* 2.6-fold in U relative to L cells only in 6-day-old colonies, despite the fact that some dividing cells are present in the very upper layers of these colonies.

## 6. Conclusions

The complement of lncRNA classes (MUTs, CUTs, etc.) is cell-type-specific, implying that lncRNA expression modulates, and/or is modulated by, cell/colony differentiation. Coregulated expression of antisense-divergent lncRNA/gene pairs appears to be largely the result of bidirectional transcription of a lncRNA and a differentially expressed gene from a common start site [14, 15, 28]. Such coregulation was the most commonly observed lncRNA/coding gene interaction seen in our study of aerial-root cells of colony biofilms and of U/L cells from smooth colonies, also during age-related differentiation. On the other hand, negative regulation of a coding gene by a lncRNA commonly occurs in the antisense-overlapping orientation. Potential negative regulation of gene expression by antisense-overlapping lncRNAs was most commonly seen in differentiated cell subpopulations, that is, upper and lower cells of 6- and 15-day-old smooth colonies, and changes most over time (between 6 and 15 days) in upper cells. Some potential negative regulations in upper versus lower cells are common to 6-day- and 15-day-old smooth colonies, but few are shared by smooth colonies and colony biofilms, which is consistent with the different lifestyles of these two types of colony populations. Fundamental processes targeted by lncRNA-negative regulation have well-established roles in ageing and differentiation, such as meiosis/sporulation, the cell cycle, cell signalling, ribosome biogenesis, translation, and cell wall assembly/maintenance. Negative regulation of these processes by lncRNAs can enable their fine-tuning during development of yeast smooth colonies and colony biofilms. Further research will be needed to prove and clarify the role of particular lncRNAs in the differentiation of cells within aging multicellular yeast populations.

## Figures and Tables

**Table 1:** Landmarks in the study of yeast lncRNA.

Discovery	lncRNA class^∗^	Strain manipulation	Technique	Reference
Cryptic Pol II transcripts		*RRP6* deletion	Microarray	[12]
Nonannotated transcripts		Wild type	Tiling array	[10]
CUT termination dependent on Nab3p		*NAB3* mutation	Microarray	[37]
Heterogenous unstable RNAs		*RRP6* deletion	Microarray	[11]
Telomeric repeat-containing RNAs (TERRAs)	*TERRAs*	*rat1-1* mutants	RT-PCR, northerns	[35]
Cryptic unstable transcripts (CUTs)	***CUTs***	*RRP6* & *TRF4* deletion	3′-long SAGE	[14]
Stable unannotated transcripts (SUTs) & CUTs	***SUTs***, ***CUTs***	*RRP6* deletion	Microarray	[15]
*PHO84* antisense lncRNA can repress in trans		Ectopic *PHO84* expression	qPCR, northerns	[59]
*PWR1/ICR1* lncRNAs and *FLO11* expression		*ΔP_FLO11_*, *cit6*, and *sfl1*	Northern blot	[41]
Condition-dependent antisense transcripts		Stationary phase, etc.	Stranded RNA-seq	[26]
Meiotic unannotated transcripts (MUTs), respiration/sporulation unannotated transcripts (rsCUTs)	***MUTs***, *rsCUTs*	Meiotic a/*α* diploids	Tiling array	[28]
Xrn1-sensitive unstable transcripts (XUTs)	***XUTs***	*XRN1* deletion	RNA-seq	[34]
*RME2* lncRNA regulates *IME2* expression		*RME2* promoter deletion	RT-PCR	[27]
CUT repression of metal homeostasis genes	*CD-CUTs*	NMD/CD-CUT mutants	Northern blot	[31]
*IME1* and *IME4* expression regulated by lncRNA		set2, set3, haploid/diploid	ChIP, northerns	[29]
lncRNA/gene pairs, coregulated in colonies		GFP-tagged *ATO1*	Microarrays	[66]
*Nrd1-unterminated transcripts (NUTs)*	*NUTs*	FRB-tagged NRD1	4tU-seq	[22]
Stress/Hog1p-regulated lncRNA transcription		*HOG1* deletion	Tiling arrays	[32]

^∗^Italics: lncRNA classes discovered; bold: classes discussed here in relation to smooth and biofilm colonies.
